# Balancing adaptation and innovation for resilience in healthcare – a metasynthesis of narratives

**DOI:** 10.1186/s12913-021-06592-0

**Published:** 2021-07-31

**Authors:** Hilda Bø Lyng, Carl Macrae, Veslemøy Guise, Cecilie Haraldseid-Driftland, Birte Fagerdal, Lene Schibevaag, Janne Gro Alsvik, Siri Wiig

**Affiliations:** 1 SHARE - Centre for Resilience in Healthcare, Faculty of Health Sciences, University of Stavanger, N-4036 Stavanger, Norway; 2 Nottingham University Business School, University of Nottingham, Nottingham, UK

**Keywords:** Adaptation, Adaptive capacity, Innovation, Resilience in healthcare, Quality in healthcare

## Abstract

**Background:**

Adaptation and innovation are both described as instrumental for resilience in healthcare. However, the relatedness between these dimensions of resilience in healthcare has not yet been studied. This study seeks to develop a conceptual understanding of adaptation and innovation as a basis for resilience in healthcare. The overall aim of this study is therefore to explore how adaptation and innovation can be described and understood across different healthcare settings. To this end, the overall aim will be investigated by identifying what constitutes adaptation and innovation in healthcare, the mechanisms involved, and what type of responses adaptation and innovation are associated with.

**Methods:**

The method used to develop understanding across a variety of healthcare contexts, was to first conduct a narrative inquiry of a comprehensive dataset from various empirical settings (e.g., maternity, transitional care, telecare), that were later analysed in accordance with grounded theory. Narrative inquiry provided a contextually informed synthesis of the phenomenon, while the use of grounded theory methodology allowed for cross-contextual comparison of adaptation and innovation in terms of resilience in healthcare.

**Results:**

The results identified an imbalance between adaptation and innovation. If short-term adaptations are used too extensively, they may mask system deficiencies and furthermore leave the organization vulnerable, by relying too much on the efforts of a few individuals. Hence, short-term adaptations may end up a barrier for resilience in healthcare. Long-term adaptations and innovation of products, processes and practices proved to be of a lower priority, but had the potential of addressing the flaws of the system by proactively re-organizing and re-designing routines and practices.

**Conclusions:**

This study develops a new conceptual account of adaptation and innovation as a basis for resilience in healthcare. Findings emerging from this study indicate that a balance between adaptation and innovation should be sought when seeking resilience in healthcare. Adaptations can furthermore be divided into short-term and long-term adaptations, creating the need to balance between these different types of adaptations. Short-term adaptations that adopt the pattern of firefighting can risk generating complex and unintended outcomes, but where no significant changes are made to organization of the system. Long-term adaptations, on the other hand, introduce re-organization of the system based on feedback, and therefore can provide a proactive response to system deficiencies. We propose a pattern of adaptation in resilience in healthcare: from short-term adjustments, to long-term reorganizations, to innovations.

**Supplementary Information:**

The online version contains supplementary material available at 10.1186/s12913-021-06592-0.

## Background

Resilience in healthcare (RiH) is a relatively new field of research which has gained increasing interest in healthcare studies as a way of understanding quality and patient safety [1, 2]. As such, theory building is necessary to establish a common foundation for this field of research to further grow.

There is consensus among scholars across disciplines that adaptation is instrumental for building RiH [2]. In terms of healthcare, adaptation is found so fundamental, that it makes up the cornerstone in the definition of resilience in healthcare; *“the capacity to adapt to challenges and changes at different system levels, to maintain high quality care”   * [2:6] . This definition underpins our understanding of resilience in this paper, which is viewed as a continuous process for obtaining quality care and patient safety.

However, despite the necessity of adaptation when facing variability and complexity in healthcare systems, not all adaptations enhance resilience [3] and our theoretical understanding of adaptation in healthcare remains relatively underdeveloped.

A necessary first step for building our understanding of adaptive capacity in healthcare is to distinguish the terms *adaptive capacity* and *adaptation.* These terms are sometimes used interchangeably in the literature. However, while adaptation refers to a specific mechanism or action in response to a particular challenge or change, adaptive capacity refers to the underlying ability of a system, team, or organization to perform adaptations [4]. As such, an individual healthcare professional adapting their own practices, is not necessarily the equivalent of building adaptive capacity within an organization. Correspondingly, an organization may possess a high level of adaptive capacity even though no adaptations are taking place.

The second step is to acknowledge the double-sided nature of adaptations. Resilience in healthcare does not increase linearly with the number of adaptations taking place. Adaptations can have both positive and negative effects for individuals and organizations at different levels [5]. We therefore need to develop a refined understanding of adaptations in healthcare contexts, so that adaptations with positive organizational outcomes can be identified and favoured at the expense of maladaptation. Furthermore, an adaptation that is successful today may not be successful over time, and a successful adaptation at one organizational level (micro, meso, macro) may turn out to be maladaptive at a different organizational level [4]. The resilience in healthcare literature has not yet dealt with this phenomenon in any detail.

Jones and Levine [6] argue that *“A key characteristic of adaptive capacity relates to the system’s ability to foster innovation and support new practices”*. However, the relationship between adaptation and innovation, and their influence on resilience in healthcare is yet to be described, even though both adaptations and innovation are related to quality and change in healthcare [5, 7, 8].

Innovation depends upon a full process, from idea to implementation, in order to succeed [8]. This posits a difference between adaptations and innovation in the amount of resources needed. A question to be raised is therefore how extensive adaptations need to be, in terms of change, to be defined as an innovation? In the traditional innovation literature, derived from product development, innovation refers to technology which can be observed and agreed upon. However, service innovations take the form of changes in practices and in relationships between stakeholders, and the distinction between adaptations and innovations are therefore more ambiguous. As such, innovation in public healthcare settings often use different types of processes and the innovations are developed to meet different objectives [9]. Hartley ([10]:27) suggests the following definition for innovation in healthcare: *“innovations need to be perceived as new by a proportion of key stakeholders”*.

Innovation is traditionally described as re-combinations of already existing knowledge and is often viewed through a knowledge lens in the literature [11–13]. This “knowledge perspective” is almost lacking in ideas about adaptation, which typically is viewed through a practice lens, where contextual experience is emphasized for understanding how healthcare professionals do their everyday work and for aligning differing demands [7, 14].

Furthermore, adaptations may introduce a dilemma, in terms of innovation, for resilience in healthcare. On the one hand, adaptations provide valuable inputs to the innovation process [10]. On the other hand, adaptations introduce a barrier for innovation in healthcare, where the ability to adapt, even to worsening conditions, reduces the motivation to innovate [15].

### Aim

The overall aim for this explorative study is to develop a new conceptual understanding of adaptation and innovation across different healthcare settings. We will address this aim by identifying what constitutes adaptation and innovation for resilient healthcare, the mechanisms involved, and what types of responses that adaptation and innovation are associated with?

### Theoretical framework

#### Resilience

Resilience is a term used across several disciplines and traditions. In terms of healthcare, resilience is particularly influenced by theory from social ecology, resilience engineering and psychology [2, 16]. Resilience engineering, in its focus on the ability to “bounce back” to some form of equilibrium state, contributes understanding of how individuals support the adaptation of complex socio-technical systems (e. g [17, 18].). Theories of psychological resilience focuses on the individual, and the ability of individuals to cope and grow in terms of challenges like stress and trauma, ideas which may be applicable to individuals working at the front-line of healthcare [19, 20]. In terms of ecology, the focus is directed towards responses of readiness, responsiveness, and recovery to external disruptions [21–25]. In social ecology, adaptation for resilience is described as a continuous cycle of growth, conservation, release and renewal [23]. In this perspective, resilience is seen to decrease during the conservation phase, were a system becomes more brittle, and expands as a system shifts into the renewal and the growth phase.

Despite being situated in different traditions, there are common elements between traditions in understanding resilience, such as: the ability of individuals, organizations and systems to bounce back to an equilibrium phase after a disruptive event, the ability to adapt when faced with pressure and challenges, and some form of reorganization or revitalization as a response to the disruption [2, 26]. The field of resilience in healthcare literature seeks to address resilience at micro, meso and macro levels, and so input from different traditions is useful to provide an overall understanding.

However, if the resilience term is used to broadly, there is a risk of it becoming a “one size fits all” concept and, too vague to be operationalized or useful. One particular gap in the RiH literature concerns the theory of the role and nature of adaptation and innovation in resilience, even though both processes are often described as fundamental.

#### Innovation and adaptation

Like the concept of resilience there also exist different concepts and models for understanding innovation and adaptation across traditions. However, many of these are engaged with similar processes and activities, like quality and change in healthcare, which are central concepts for resilience in healthcare [5, 7, 8]. In the following, key theoretical contributions are presented, describing different ways of understanding adaptation.

For organizations to succeed, they need to adapt in accordance to changing conditions. When interdependencies change, there is a need for coordination mechanisms to change accordingly, situations which Grote et al. [27] define as adaptive coordination. Through their conceptual paper the authors propose the duality between stability and flexibility to act as triggers for adaptation [27].

Flexibility has typically been viewed as a facilitator for agile, creative, and innovative teams. However, recent research provides evidence that agile and innovating teams perform better when high flexibility is coupled with stability mechanisms. Adaptations seeking to develop stability are based on predictability, reliability, and efficiency requirements, while adaptations facilitating flexibility allow for variability in a range of practices and processes [27]. The understanding of adaptations as a tactic to achieve both flexibility and stability mechanisms, indicates that research concerning adaptations needs to embrace this duality and develop a refined empirical understanding of how some adaptations facilitate stability and others flexibility.

In their exploration of resilience in maternity care Macrae and Draycott [28] describes two different types of adaptations: dynamic adjustments and adaptive reorganizations. Dynamic adjustments refer to moment-by-moment adjustments, accommodations, and responses to variations in practical work. Adaptive reorganizations refer to effortful processes of reflection, inventions, and adaptions, and are put into work for the objective of reorganizing and redesigning practical work and organizational systems due to disruptive and unexpected events.

A similar way of categorizing adaptation is proposed by Löf [24] who separates adaptability into adaptations and transformations. Adaptation refers to adapting behaviours to disturbance and change and refers to, for instance, situations where healthcare professionals compensate for deficiencies within a system and within the institutional boundaries. Adaptation is aimed at *maintaining a current system regime*, like when a team member takes on extra responsibilities, due to a colleague on sick leave, to keep the system functioning as normal. Adaptations are at risk of producing complex and unintended consequences due to this fire-fighting behaviour, where the system is kept as is [24]. Transformations refers to the *changing of the system configuration*, and therefore the navigation of a system from an undesirable, but self-reinforcing, regime to another ([24]: 531).

Even though adaptation is found to be a valuable tactic for ensuring resilience in healthcare, researchers also describe a dual role and nature of adaptations [3, 5, 27, 29, 30]. Branlat and Woods [3] found three patterns of maladaptation; firstly, decompensation where the system is overloaded and there is no capacity left to perform adaptations. Secondly, working at cross-purposes - which refers to situations where sub-systems or roles present behaviour that proves locally adaptive, but maladaptive at the system level. And thirdly, adaptive behaviour may be trapped in outdated behaviours in a form of “never change a winning team” mentality.

The resilience in healthcare literature purposefully draws on various resilience traditions. As such, there is a need to develop more precise and context-specific definitions, understandings and operationalizations of key aspects of resilience in healthcare, like adaptation and innovation. However, this process needs to be sensitive to the underlying values, perspectives, and assumptions, when importing relevant concepts in healthcare in order to develop the theoretical field [31]. Research in the RiH field has been criticized for being insufficiently grounded in empirical data in its theoretical development, which risks the uncritical adoption of concepts from other disciplines. It is therefore important for new theoretical development to be based on empirical data in order to provide contextual understanding of what is demonstrated rather than assumed.

## Methods

### Contextual setting

This study uses data from various healthcare settings within the context of Norwegian healthcare system. The Norwegian health system is a universal, nationalized healthcare system, where the specialised healthcare services are organized in four regional health authorities. The municipalities are responsible for providing primary care services to their inhabitants. The Norwegian healthcare system is publicly funded, and figures show that Norway spent 10,4% of its GDP on healthcare in 2017, with public sources accounting for 85,5% of the health expenditures [32]. All data included in this study are from public hospitals and public primary care settings and therefore relate to non-profit institutions only.

### Design and sample selection

This study is part of the Resilience in Healthcare (RiH) research programme which applies a collaborative interactive research design aiming to establish a RIH framework including theoretical and practical outcomes (2018–2023) (see Aase et al. [33] for the full study protocol). The RiH programme has two main phases – an explorative phase with screening, synthesising, and validation of results from a sample of existing empirical projects covering a variety of healthcare settings; and an intervention phase that includes design, implementation and evaluation of measures to facilitate resilient capacity in healthcare systems ([33]:4–5).

In this article, we report findings from the exploratory phase. In the exploratory phase, the research programme uses data from a sample of research projects from multiple empirical healthcare settings, across all levels of the healthcare system (micro, meso, macro). The sample is selected from several former and ongoing research projects involving members of SHARE, the Centre for Resilience in Healthcare in Norway. The selection process involved the screening of a total of 50 research projects (including research projects, post-doctoral projects, and PhD projects) according to an established screening protocol (please see Aase et al. ([33]:6–7) for detailed info) and a Quality and Resilience Trigger Tool (please see Aase et al. ([33]:6–7) for detailed info). The purpose of this screening process was to establish how the projects related to resilience and which quality components they covered. The screening process resulted in a sample of 25 projects that were selected for inclusion to secure a comprehensive range of empirical healthcare settings (e.g. homecare, nursing homes, hospital, prehospital critical care), stakeholders (e.g. next of kin, patients, users, healthcare professionals, managers, regulators), quality dimensions (patient safety, clinical effectiveness, patient centredness, coordination), and adaptive capacities (individual, team, unit, organisational, larger system) ([33]:5)]. The screening of relevant projects to be included was performed and agreed upon by all authors involved in this research project.

From the sample of 25 included projects, we selected a total of 14 projects for inclusion in the study reported on in this article (see overview in attachment 1). The selection criterion was that the projects had to have produced empirical articles that could be used as data material for the analysis. The objective for the research undertaken in this specific article is to develop understanding of resilience in healthcare, and more specifically how adaptation and innovation can be described and understood across different healthcare settings.

### Data collection

The 14 included research projects in this study have published 22 peer reviewed articles and book chapters and 6 PhD theses (each thesis included 3 articles and a synopsis). For full details of the included projects see attachment 1. The text produced in the publications constitutes our data material and was collected from the journal web sites, databases, or from the publicly available databases over Norwegian PhD theses. The data collection took place from February 2020–September 2020.

### Data analysis

The analytical process was a metasynthesis of narratives from the 14 projects. This process started by writing a narrative from each of the included projects. All narratives were developed in researcher pairs according to a predefined template agreed upon in the research team. The narrative development covered the following dimensions based on Macrae and Wiig [34]:

1. Defining the phenomena of resilience (ca 150–200 words on each question):
  1. Resilience for what? (What goals and objectives are resilience supporting?)
  2. Resilience to what? (What triggers, activates or necessitates resilience?)
  3. Resilience of what? (What materials and resources underpin resilience?)
  4. Resilience through what? (What mechanisms, activities and interactions enact resilience?)
2. Describe settings, system level, staff involved, professions, competence level, and contextual conditions of where the project takes place.
3. Resulting in a 4–7 pages narrative per project.

The finished narratives from the 14 projects totalled 70 pages and represents a wide variety of healthcare contexts, tasks, and levels. The narratives were developed by pairs of in researchers and discussed and refined in several iterations among the project team.

The narrative inquiry methodology allowed for a contextually informed synthesis of a comprehensive dataset [35, 36]. The contextual setting was given emphasis in all narratives, with an extensive use of original quotes, so that the voice of the empirical data was captured. As such, narrative inquiry was used to set words to themes of resilience in the dataset, as the majority of the included projects used different theoretical frameworks as a foundation for their discussion. The researcher pairs, developing the narratives, therefore had to interpret the data to discover their relevance for informing resilience and adaptive capacity. In this paper, where the aim was to explore how adaptation and innovation can be described and understood across different healthcare settings, all narratives were uploaded in Nvivo 13 to support structure and document the analysis process. To guide the analysis the following analytical questions (AQ) were used:

1. AQ1: What constitutes adaptation and innovation across healthcare settings?
2. AQ2: What type of mechanisms relate to adaptation and innovation across healthcare settings?
3. AQ3: What type of resilient responses are adaptations and innovations associated with?

**Fig. 1:**
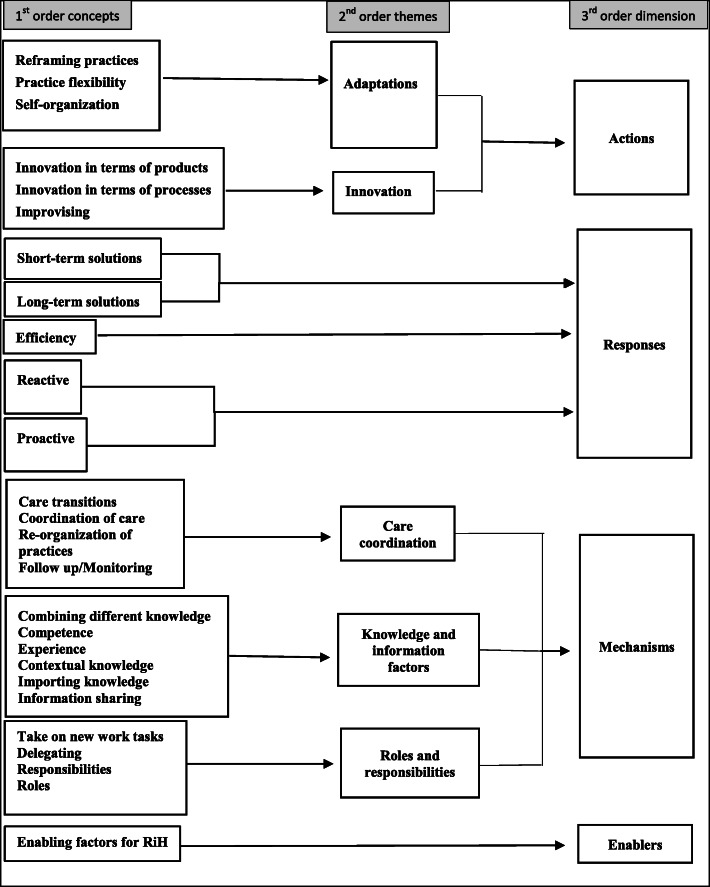
Data structure model [37]

The data from the narrative metasynthesis was analysed inductively according to grounded theory as described by Gioia et al. [37], from 1st order concepts, to 2nd order themes and 3rd order dimensions, see data structure model in Fig. [1]. The initial coding, constituting the 1st order concepts, included concepts emerging directly from the data. These initial 1st order concepts were further aggregated into 2nd order themes and 3rd dimensions, where aggregation included informing theory and abstraction. All authors met at a regular basis to discuss the aggregation process.

The analysis was performed in the following steps: Firstly, addressing AQ1, 1st order mechanisms found to be ways of handling variability and complexity, were aggregated into the 2nd order themes adaptation and innovation. Adaptation referred to instances of adapting behaviours in response to disturbance and change [24] and innovations referred to something new that was developed into a process or product [38]. *Reframing practices, practice flexibility,* and *self-organization* constituted the 2nd order theme adaptations, while *product innovations, process innovations,* and *improvisation* (idea in development) constituted the innovation term. Adaptations accounted for 74 instances and innovation accounted for 6 instances. Secondly, addressing AQ2; findings from AQ1 were cross-tabulated against mechanisms, using the matrix function in the NVivo 12 software. Thirdly, to address AQ3, the findings from AQ1 were cross-tabulated against the third order dimensions; responses and enablers, see Fig. 1.

Combining different qualitative methodologies is an increasingly common research strategy. However, metasynthesis where one takes advantage of narrative inquiry and grounded theory has not been used extensively. Arising from American pragmatism, both these methods share a similar history, even if their approaches differ in terms of their view of the researcher, phenomenon, interpretation, and analysis. Lal et al. ([35]:16) explored the potential for combining grounded theory and narrative inquiry and found this combination to be “*theoretical commensurable; they can be natural allies within a qualitative study”*. Like Lal et al. [35] note, this combination of methodologies allowed for complementary understanding within this synthesis. The narrative inquiry allowed for synthesis of a large and diverse dataset, while emphasizing contextual aspects. The use of grounded theory provided an ability for inductive cross-contextual comparison of important aspects for adaptation in terms of resilience in healthcare.

## Findings

Adaptation is a response to a misalignment between demands and capacities, and where demands may be of internal or external origin. Internal demands in this cross-contextual healthcare setting were based on a lack of resources (like staff, competence, experience, equipment and technology, access to appropriate knowledge, information sharing, involvement, and communication, and disruptions of processes). Demands were also introduced from external sources. Budget cuts and regulatory demands from the municipality and national health authorities were found to trigger adaptations.

**Fig. 2:**
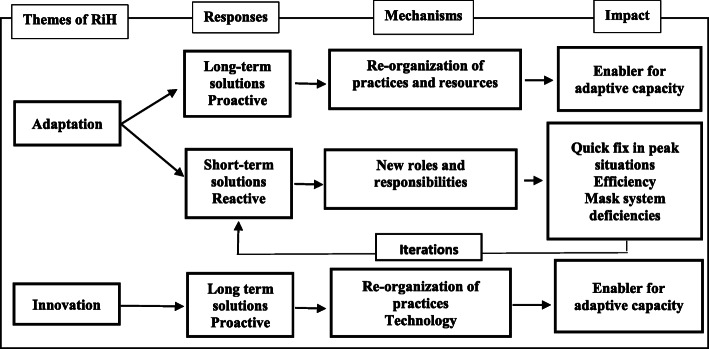
The impact of adaptation and innovation for resilience in healthcare

In order to cope with these external and internal demands, healthcare professionals had to broaden their view to seek ways of coping. To do so, healthcare professionals could choose ways of adapting their practices and processes or they could create product or process innovations. The findings from AQ1 showed a strong imbalance between instances of adaptation and instances of innovation. To gain a better understanding of this imbalance, a matrix between the adaptation and the innovation 2nd order themes, against mechanisms, responses and enablers was developed and performed in the NVivo 12 software. Figure 2 gives an overview of the findings from the analysis and illustrates the finding that adaptations could be grouped into two types based on their mechanisms, response, and impact.

### Mechanisms and responses found related to adaptation and innovation

#### Innovation providing long-term solutions

The results from the matrix showed that innovations included both product and process innovations, and all instances within this dimension were related to long-term solutions. Innovations were mostly referred as the changing of practices and processes, which meant a re-organization of practices.

Furthermore, innovations in healthcare services were also found to include new technology that was introduced to ease the workload. However, the implementation of new technology also required healthcare personnel to adapt their traditional practices and procedures. This is illustrated in the following example, where physicians used mobile devices to increase efficiency (not needing to go to the staff room where the computer was located) and further for availability concerns (checking the medication while still communicating with the patient). Even though mobile devices provided an innovative solution for the medication administration process, the environment was not necessarily rigged for such technology, and access to Wi-Fi became a problem in some areas of the nursing home. This example therefore illustrates the close relationship between innovation and adaptations in healthcare settings, where the outcome is not strictly innovation or adaptation, but involves both aspects.

*The use of mobile devices with medication administration record functionalities were found to improve quality in the medication administration process in both ordering and preparing phases. However, in order to use such mobile knowledge sources, the users needed to have access to Wi-Fi, which sometimes posted a challenge. (Included in project 14, see attachment 1).*

#### Adaptation providing long term solutions

Two thirds of the adaptations found in the dataset were associated with long-term solutions, and the majority of long-term solutions was related to the re-organization and coordination of practices. This is exemplified in the following quote where a surgeon had invented a surgical procedure and continued to use this new procedure in all his surgeries, to ensure patient safety. This personal technique illustrates how a procedure for entering the vein is “transformed” into a tacit ability for anticipation and handling future events of this kind, such as the patient turning ill and the vein access becoming more difficult. The quote furthermore illustrates the potential of adaptations as further input for developing innovations.

*“It is partially a craft… the basic principles are necessary, but techniques can be adapted to achieve the same goal. For example, during a procedure where entering of a needle is involved… I use to mark the skin with the hollow end of a pen, to ensure that when a swelling occurs the mark will still be there, and I will not need to “feel” [my way to the artery] again when I enter the needle. [This is also important] when the pulse gets weak, the patient is ill, and you do not know where the artery really is.” (Included in project 6, ).*

Another element found important for adaptations leading to long-term solutions, was to re-organize for having appropriate resources available in care coordination. This could be to ensure buffer resources (first example) or by designating knowledgeable people to specific resource roles (second example).

*The city-based maternity services had established a coordination centre consisting of a pool of employees with no departmental affiliation, but they were allocated according to a resource needs principle. To enhance flexibility and ensure available resources in the maternity service the coordinating centre consisted of several midwives and nurses prepared to start the day at one ward but could be reallocated to another if capacity or expertise needs indicated that. (included in project 1).*

*In this homecare service, resources were reallocated to create a new position for a dementia coordinator, as prescribed in a new national guideline that will be implemented in 2020 (`dementia 2020`). (Included in project 3).*

Another form of re-organizing resources for achieving long-term outcomes was to organize for staff continuity. The appointment of, in this quote, surgical personnel to specific units are decisions made by managers at the meso-level. However, the findings gained by this enabler of adaptive capacity took place at the micro-level.

*Another compensating system factor was that operating personnel were exposed to only one section, which over time boosted specialized knowledge, confidence levels, and the ability to become proficient with the equipment and select the right equipment at the right time. (Included in project 6).*

#### Short term adaptations

The remaining third of the adaptations consisted of short-term solutions. These quick fixes mostly accounted for situations where individuals took on additional work tasks and new responsibilities to ensure quality of care. This form of adaptation meant that some individuals compensated for system deficiencies, making the organization dependent on these individuals to function well—which in turn created a source of vulnerability. Short-term adaptations are not intended to permanently change the system, and new actions will be required in the future to tackle similar challenges, hence that additional arrow illustrating an iterative pattern in Fig. 2. An example is in the first quote below, where a healthcare professional, due to guidelines, was not present to perform his duties based on his late shift the previous day, meaning another healthcare professional had to take on extra responsibility to cover the absence and to keep up with the original surgical schedule. In the second quote below, the manager in a nursing home describes the challenges of having limited resources, and that on one shift, a single nurse ended up with the responsibility for 130 patients.

*“The reason for the “slip” at the last check point, the 1st nurse anaesthetist explains, was due to a late shift the night before that had resulted in one individual being unable to assume his day shift (the individual has an 11 h quarantine time). The following shift then became one individual short. As a consequence, one person on this shift became responsible for two patients simultaneously. The nurse believes that such situations increase the workload and stress levels, which can lead to mistakes”. (Included in project 6).*

*Sometimes department managers performed nursing duties during the day shift, or one nurse assumed responsibility for approximately 130 patients across seven departments. (Included in project 3).*

#### The impact of adaptation

Long-terms solutions, whether in the form of adaptations or innovations, were found to be associated with the code *enablers for adaptive capacity*. These enablers consisted of proactive solutions of adaptation that included factors like the organization of resources, the distribution of knowledge, the development of trust, and for easing communication.

Short-term adaptations accounted for ‘fire-fighting’ behaviour in peak situations (reactive actions), revealing only short-term benefits, even though they were of immense value at that specific point in time. When seen in a longer perspective, these short-term adaptations were found to mask system deficiencies, when not reported from the micro-level to the meso and macro-level, thereby ensuring management remained unaware of the challenges taking place.

## Discussion

Findings showed that long-term adaptations were highly associated with new ways of organizing practices, while short-term adaptations accounted for situations where healthcare professionals took on additional responsibilities and work to ‘keep the wheels turning’. Taking on additional tasks and responsibilities can be noble responses to support care quality and resilience in healthcare services to cope in peak and unexpected situations. However, if used too extensively, short-term adaptations can mask underlying deficiencies of the system and thereby end up as barriers for systemic resilience in healthcare [5, 28, 39]. The meso and macro level is left ignorant of micro-level deficiencies, hiding problems and creating the erroneous perspective that healthcare services are performed satisfactorily with the available resources—a situation which can lead to major organisational failure [40, 41] .

Löf [24] described adaptive firefighting behaviour to be at risk of producing unintended consequences, as these short-term adaptations only provided a situational effect, leaving the organization otherwise unchanged. Branlat and Woods [3] furthermore argue that short-term adaptations are at risk of working at cross-purposes, where the adaptation is successful at the micro-level, but unsuccessful at the meso and macro-level. Short-term adaptations may also pose a risk for maladaptation in the form of outdated behaviour, if the same response is used over and over, without reorganizing the system.

Adaptations leading to long-term solutions were mostly related to the organization of practices, and thus reflect the practice-oriented nature of adaptive capacity in healthcare services. Long-term adaptations in these settings suggest that adaptations often consist of thoughtfulness and evaluation, open to learning, when handling traditional everyday tasks, instead of “out of the box” solutions [8]. The low level of innovation further reflects this understanding. However, long-term adaptation may also be at risk of maladaptation in the form of outdated behaviour [3], if the reframing and reorganization are not based on reflection, learning and new perspectives, and instead falls into a pattern where old solutions are transferred and implemented to solve new challenges [42, 43].

### From adaptation to innovation

How do innovation and adaptation differ? Innovation is defined as a full process that can be managed and organized [38], and does not therefore simply consist of short-term activities or creative ideas. Short-term adaptations may only affect a single healthcare worker (e. g. in taking on extra responsibilities), however long-term adaptations and innovations usually affect an entire team, organization, management, or the whole system [44]. For ideas to be developed into innovations, they need to go through a process from idea, development, and implementation. Also, a part of the innovation process is the diffusion of the innovation, to other organizations or systems, a feature not necessary for adaptations, which can be more localized (“the way we do things here”). Based on the above, innovations are for the most part, more time and resource demanding than adaptations. However, several adaptations within this dataset may have the ability to be turned into innovations, if further developed.

In terms of resilience, it is important to notice that there is no correlation between the level of change and the level of resilience. Radical re-organizations or innovation do not necessarily lead to an increase of resilience, and vice versa where incremental changes can be fundamental enablers for resilience [26].

Innovations are mainly re-combinations of already existing knowledge [12, 13] thereby relying on the import of new knowledge to develop novel combinations, a feature not necessarily needed for making adaptations. Even though both adaptations and innovations refer to long-term solutions and furthermore were found to enable adaptive capacity, the low level of external import of new knowledge, with the purpose of developing innovations, was noticeable. Furthermore, innovations mostly happen at the boundaries between disciplines and specializations [42], meaning that innovation is facilitated by the combining of different knowledge, perspectives, and specializations [11]. Innovation therefore requires time, effort, and resources in early phases to capture and converge different perspectives and knowledge into innovations.

Correspondingly, short-term adaptations represent the opposite, where adaptations are needed to put out the situational fire. Even though both short-term solutions and long-term solutions are important, our findings indicate that there is a need in healthcare settings to balance these different types of adaptation and not make short-term adaptations as default responses. Van de Ven [15] describes an innovation barrier among individuals and organizations to be an unconscious adaptation to a slowly changing environment, even if the changes are in terms of worsening conditions. He further argues that the threshold for innovative action is often not triggered if the actors are adapting to changes over time ([15]:595). This understanding may provide an explanation for the low level of innovation taking place across these healthcare settings, where limited resources are allocated for developing innovations.

Rodin [25] addresses the phenomena of resistance to implementing new practices and products, also described as the not-invented-here syndrome in innovation literature [45], which can be caused by unfamiliarity. She further states that such problems can be avoided by repurposing existing programs and practices. This may be especially beneficial in situations in need of a rapid solution and where a large number of people are involved, as often is the case in different types of systems in healthcare, such as emergency departments and intensive care units.

Another explanation for the low level of innovation may be a disproportionate time and effort used at the different adaptation phases described by Frick et al. [29]. Where short term adaptations relate to moment-by-moment [28] responses (responding phase) to internal and external demands (cues detected in the recognition phase), less resources are put into the reframing (developing a shared understanding for re-organization of practices) and reflecting phase (reflecting on the adaptation outcome as input for reorganization). The development of long-term adaptations and innovations therefore rely on management to direct resources to all these phases, to embrace and fertilize ideas from the front-line actors, and to report short-term adaptations upwards in the system for further macro-level reflection.

### Learning

Another disadvantage of being too dependent on “quick-fixes” is the distribution of learning. Short-term adaptations were represented by individuals taking responsibility for new tasks; hence these individuals learned to perform new tasks which later increased their competences and skills. The wider organizational learning effects of such short-term adaptations are therefore mainly absent, and the organization continues to depend on specific champions taking on additional responsibilities to ensure patient safety. Learning is therefore unevenly distributed across team members, creating a situation where some individuals end up knowledgeable champions within a vulnerable organization. Short-term adaptations may thereby imply individual learning at the expense of organizational learning.

Furthermore, short-term adaptations are based on single-loop learning and the continuous adaptation of actions to reach the desired outcome, while long-term adaptations by their questioning of the fundamental challenges involve double-loop learning [46, 47]. As illustrated in Fig. 2, short-term adaptations do not normally have a permanent impact, as there are minimal changes to the system, but rather result in an iterative pattern where short-term solutions are required as responses over and over again.

### Adaptations for stability and flexibility

Systems seek stability, which often presents itself as a desire to continue doing things as they always have done [25, 48]. However, the use of rigid control mechanisms for ensuring stability often end up eroding resilience. Hence stability is not enough to provide resilience on its own, and flexibility and learning are also needed [21].

Grote et al. [27] note the importance of adaptations to provide both stability and flexibility, and furthermore to find a balance between these elements in the specific organization. Long-term adaptations were found in our study to provide a tactic for developing stability, through their ability for adaptive reorganizing and re-coordination. The same potential holds for innovation. In terms of flexibility, both short-term and long-term adaptations were facilitators, but in different ways. For short-term adaptations, some individuals proved highly flexible when they took on new tasks and responsibilities. For long-term adaptations, flexibility was facilitated by adapting the coordination of practices in more efficient ways. This means that long-term adaptations, by providing stability (thereby providing efficiency), can – somewhat paradoxically – create the space for healthcare professionals to act more flexible.

### Dynamic adjustments and adaptive reorganization

Macrae and Draycott [28] described dynamic adjustment and adaptive reorganization as two separate concepts. These dimensions possess similarities to those of the role of short-term and long-term adaptations found in our research, but also some contradictions. Short-term adaptations and dynamic adjustments are both moment-by-moment adjustments to variations in practical work and both are reactive responses. However, dynamic adjustments are exemplified as organizational adjustments, while short-term adaptations in this setting were mostly individual efforts to compensate for diverse challenges such as sick leave, peak situations, and a lack of competence.

Adaptive reorganizations are described as reorganization and redesign in terms of disruptions and unexpected events [28]. Unexpected situations (coded in the dataset as *handling the unexpected*) in our study was tackled by either an individual compensating (e.g., healthcare professionals took on more responsibilities) or by the whole team being willing to contribute their efforts to solve the challenge. Hence, the actors did not engage in redesign or reorganization of their practices. In terms of disruptions (e.g., introducing new technology) or in the reorganization of long-term practices and processes, the findings within this study comply with the term adaptive reorganizations described by Macrae and Draycott [28]. Based on the knowledge gained by combining the findings from this study, with the Macrae and Draycott [28] study, one might suggest that adaptations follow a pattern from short-term *adjustments* (responsive quick fixes), to long-term *reorganizations* (proactive reorganizations and redesigning of routines and practices), to *innovations* (where solutions are fully developed and disseminated across organizations).

### Strengths and limitations

The novel choice of methods used to develop conceptual understanding of adaptation and innovation for resilience in healthcare across different settings, is a strength of this study. The choice of methods followed three analytical phases. Firstly, a consensus process for the selection of projects was used. The selection process was based upon a published screening protocol and a quality and resilience trigger tool to ensure reliability [33]. Consensus among the involved authors was a way of developing construct validity. Secondly, in order to synthesize the comprehensive dataset, narrative inquiry was chosen as a method for analysis [35]. Narrative inquiry allowed for contextual synthesis, with a focus on original quotes to capture the voice of the informants. The narratives were developed in pairs of researchers which provides validity to the outcomes. And finally, the narratives were used as data for an inductive grounded theory analysis, allowing for cross-case comparison across different healthcare settings. All authors met regularly, and consensus was developed for all phases of the aggregation and abstraction process, providing validity and reliability to this third phase of analysis. This way of combining methods of data analysis allowed for a more comprehensive understanding of adaptation and innovation across levels and settings, and is as such a way of responding to recent calls for multi-level and multi-setting research to understand resilient adaptations [49, 50].

A limitation of this study may be that only projects from SHARE (Centre for Resilience in Healthcare) in Norway were included in the study. In addition, patients as stakeholders were not included in our sample. Future research should seek to include projects across different countries, to further explore how the balance of adaptation and innovation for resilience in healthcare is acted out in cross-country studies, including the role of patients in these processes.

## Conclusion

The aim of this study was to provide a new conceptual account of adaptation and innovation as the basis for resilience in healthcare. Based on the above discussion, the establishment of a balance between adaptation and innovation is important to increase resilience in healthcare services. Findings showed that adaptations could purposefully be separated into short-term and long-term adaptations.

There will always be a need in any organization to carry out some “quick-fixes” in peak situations. However, long-term adaptations where care coordination is sought optimized through re-organization should be preferred. Short-term adaptations are, due to the fire-fighting pattern, at risk of generating complex and unintended outcomes, without making changes to the organization of the system. Long-term adaptations, on the other hand, encourage re-organization of the system based on feedback, and therefore provides a proactive response to system deficiencies.

Findings from this research show that long-term adaptations also provide flexibility to healthcare professionals, a balance which is described as valuable for ambidextrous organizations, like healthcare [51]. Additionally, innovative solutions should be sought, as the acquisition of new perspectives and knowledge may provide novel ways for solving challenges.

Combining the findings from this research, with those of Macrae and Draycott [28] study, allows for a conceptual understanding where adaptations follow a pattern from short-term adjustments, to long-term reorganizations, to innovations. By increasing our understanding of adaptations across contextual settings, this study has the potential to inform healthcare organizations, teams, and managers on how to evaluate and balance the use of adaptations and innovation.

### Implications

#### Implications for theory

This study seeks to develop a new conceptual understanding of adaptation and innovation as pillars of resilience in healthcare, and thus contributes new theory to fill some existing gaps in the literature. Firstly, this study provides new understanding of the role and nature of adaptation and innovation as keys for resilience in healthcare. Even though both have long been described as instrumental for resilience in healthcare, their role and nature are not yet fully understood. This study contributes to that end.

Secondly, the findings from this study add to the conceptual understanding that adaptations can purposefully be divided into short-term and long-term solutions, where the outcomes are fundamentally different in terms of resilience. This conceptual understanding is new to the RIH literature, even though it shares some similarities with findings in other studies and traditions [24, 25, 27, 28]. Thirdly, short-term and long-term adaptations were based on different mechanisms and expressed by different actors. Short-term adaptations were characterized by individuals taking on additional responsibilities, while long-term adaptations were more of a collective effort to reorganize practices and resources. These findings provide new conceptual understanding to the RiH field, and furthermore extends the learning literature to aspects of resilience in healthcare [46, 47].

Innovation is scarcely examined in the RiH literature, even though it is described as highly influential in the development of resilience [52]. In this study there were few instances of innovation development. In cases where innovation was found in the dataset, it further highlights a need for re-organizing existing practices. This provides a new emphasis on the interdependencies between adaptation and innovation.

#### Implications for practice

Calls have been raised for studies to develop our understanding of adaptive capacity and resilience in healthcare across contexts and levels [49, 53], in order to provide a more complete understanding of the healthcare context. However, understanding how adaptations and innovations act out across healthcare contexts and levels, may support improvement at informing the meso and macro-level of health care.

As short-term adaptations can mask system deficiencies, these short-term adaptations need to be reported upwards in the system to actors at the meso and macro-levels to inform their decision making. If reported, these short-term adaptations may provide a  potential, and, if not reported or acted on, as a barrier for resilience which is the case in this study. Front-line actors at the micro-level should be supported to not simply mask the flaws of the system over and over with short-term adaptations, but instead be encouraged by management to contribute collaborative efforts to propose and develop long-term adaptations within their organization.

A large number of short-term adaptations should provide a signal to managers to provide resources for the re-organizations of practices and innovation development. Such responses can be resource intensive in the early phase but may reveal long-term effects that promote lasting resilience and efficiency.

The existence of a large number of adaptations also indicate that front-line workers have considerable knowledge of what adaptations are needed for ensuring quality care. As such, bottom-up initiatives for innovation development, within the organization or in joint collaborations with the industry, should be encouraged. To ensure innovation and adoption in the healthcare industry that both improves care for patients and is supportive of healthcare work, innovations need to be sensitive to and practically address healthcare needs at the micro-level. That emphasises the importance of shared understanding and perspective taking between healthcare workers and external innovation developers [54].

## Supplementary Information

**Additional file 1.**

## Data Availability

The datasets used and analysed during the current study are available from the corresponding author on reasonable request.

## Abbreviations

RiH: Resilience in Healthcare
SHARE: Centre for Resilience in Healthcare
