# Supplementary material for: Balancing adaptation and innovation for resilience in healthcare – a metasynthesis of narratives
Source: BMC Health Serv Res. 2021 Jul 31;21:759. doi: 10.1186/s12913-021-06592-0 (PMC8325788; doi:10.1186/s12913-021-06592-0)
Supplement: Supplementary file 1 — Additional file 1. [file 12913_2021_6592_MOESM1_ESM.docx]

Attachment 1. Included projects

| **Project title** | **Year** | **Empirical setting** | **Involved Stakeholders** |
| --- | --- | --- | --- |
| **1. QUASER Quality and Safety in European Hospitals** | 2010-2013 | Hospitals | 10 European Hospitals from 5 European Countries |
| **2.Next-of-kin involvement in regulatory investigation of adverse events that caused patient death** | Finalized 2019 | Norwegian regulatory body  One county  Governor’s office in Norway | Next-of-kin  Regulatory investigators |
| **3.SAFE-LEAD** | 2016-2021 | Nursing homes  Homecare services | Managers  Healthcare personnel |
| **4.Patient participation in transitional care of older patients** | Finalized 2016 | Hospital admission, discharge, transitional care | Healthcare personnel (ambulance workers, nurses, and doctors)  Older patients |
| **5.Simulation-based telecare training for home healthcare professionals** | Finalized 2019 | Homecare services | Homecare professionals |
| **6.Safe work practices in interdisciplinary surgical teamwork** | Finalized 2013 | Hospital Surgical teamwork | Healthcare professionals |
| **7.Transitional care of the elderly from a resilience perspective** | Finalized 2015 | Hospital, nursing homes, homecare services | Healthcare professionals, elderly patients |
| **8.Safe clinical practices for patients hospitalised in a suicidal crisis** | Finalized 2020 | Psychiatric care in a Norwegian hospital | Suicidal patients  Healthcare professionals |
| **9.Next-of-kin involvement in hospital cancer care** | Ongoing | Cancer department in two hospitals. Managers and staff. | Next-of-kin  Healthcare professionals  Managers |
| **10.Exploring hospital readmissions from the primary healthcare service** | Finalized 2020 | The interface between primary and secondary care. Hospitals, nursing homes. | Physicians, nurses and nursing home managers. |
| **11.Impact of active implementation of the Norwegian Musculoskeletal guideline on the use of non-traumatic musculoskeletal imaging** | Finalized 2020 | Two municipalities in Norway. Hospital and general practitioners | GPs, radiologists, radiological fellows |
| **12.Safe use of telecare for older adults in homecare services** | Ongoing | Six community homecare services in two Norwegian municipalities. | Homecare professionals (nurses and occupational therapists) |
| **13.Exploring links between resilience and macro-level development of healthcare regulation** | Ongoing | Employees at the ministry, the directorate, and the Norwegian board of health supervision | Actors at the macro level at Governmental regulatory bodies. |
| **14.A human factor approach to medication administration in nursing homes** | Finalized 2020 | Nursing homes | Healthcare personnel in nursing homes involved in medication administration |
